# Posterior tibial slope in the Emirati population: The slope is over 12 degrees

**DOI:** 10.1051/sicotj/2026048

**Published:** 2026-08-07

**Authors:** Jesper Fritz, Luke V. Tollefson, Zakee Azmi, Sebastien Parratte, Robert F. LaPrade

**Affiliations:** 1 International Knee and Joint Centre Sheikh Fatima Bint Mubarak St, Opposite Burjeel Hospital Abu Dhabi UAE; 2 Twin City Orthopedics Edina Minnesota USA

**Keywords:** Anatomical tibial axis, Mechanical tibial axis, Posterior tibial slope, Proximal anatomical tibial axis, Emirati population

## Abstract

*Introduction*: Posterior tibial slope (PTS) has been reported to be an important risk factor for anterior cruciate ligament (ACL) reconstruction (ACLR) failure. PTS varies between populations, and a mean value in the Emirati population has not been described. To evaluate PTS and to improve clinical decision-making regarding surgical thresholds for slope correcting osteotomy, a mean value for the Emirati population needs to be defined. *Methods*: This was an institutional review board-approved retrospective study of 213 consecutive Emirati patients with knee complaints visiting a single orthopedic center in 2025. Inclusion criteria were patients >18 years of age visiting the center for the first time with no previous surgery or fracture in the affected knee. Medial and lateral PTS were measured on long weight-bearing lateral tibial radiographs using the proximal and long anatomical tibial axis and mechanical tibial axis. *Results*: A total of 213 patients, 73 women and 140 men, were evaluated with a mean age of 46.0 years. The mean medial PTS was 12.8° ± 2.9° (range: 3.7–19.7°), and the mean lateral PTS was 11.9° ± 3.2° (range: 3.9–20.1°) using the proximal tibial axis. Using the long anatomical tibial axis, the mean medial PTS was 14.7° ± 3.3° (range: 4.8–25.5°), and the mean lateral PTS was 13.8° ± 3.7° (range: 5.1–24.8°). Using the mechanical tibial axis, the mean medial PTS was 12.7° ± 3.1° (range: 2.7–19.7°) and the mean lateral PTS was 12.2° ± 3.1° (range: 3.8–20.1°). Sex did not influence the PTS value (*p* > 0.05). *Discussion*: In this Emirati population, the mean medial PTS was 12.7–14.7° and the mean lateral PTS was 11.9–13.8° for the differing measurement techniques. These values are higher than other reported populations and can be used as a reference when evaluating knee injuries and contribute to surgical decision-making in ACL-deficient knees.

## Introduction

The role of the posterior tibial slope (PTS) in knee kinematics and stability is crucial [1]. Increased PTS results in a higher load on an anterior cruciate ligament (ACL) reconstruction (ACLR) graft [1] and increased overall anterior tibial translation (ATT), thereby presenting an increased risk of ACLR graft failure [2, 3]. Even though modern techniques for ACLR demonstrate excellent results, there is a reported failure rate of up to 7–20% [4, 5], where increased PTS has been reported to be an independent risk factor for ACLR failure [6].

Very few studies reporting ACLR outcomes in the Middle East are published, but a recent study from Saudi Arabia shows a return to sport rate of only 44% [7]. The reasons for this are unknown, but since PTS varies in different populations [8, 9], where Asian and Middle Eastern populations seem to have higher PTS than others [10–12], one possible reason could be persistent laxity due to increased PTS and the resultant ATT.

Over the last decade, more attention has been given to the PTS when it comes to revision ACLR, as there is an opportunity to perform a slope-reducing osteotomy to decrease the PTS in combination with a revision ACLR to lower the risk of further failures [13]. However, decision-making for when to perform such an osteotomy is not clear, but the most common indication is for a revision ACLR with a PTS >12° [14]. Even though the agreement regarding 12° as a “cut off” value for considering osteotomy is strong, how to measure the slope on plain radiographs is still debated, and several studies comparing different measurement techniques demonstrate significant differences between the measurement techniques, but good correlation within a technique [15]. Furthermore, different imaging modalities report different values for both computer tomography (CT) and magnetic resonance imaging (MRI) [16]. The most common technique is to measure the angle between the proximal tibial anatomical axis and the medial tibial plateau [17, 18] on a short lateral radiograph. However, recent studies suggest that the tibial mechanical axis and medial tibial plateau on long lateral radiographs are more accurate [19].

A mean PTS value in the Emirati population has not been described, and since there is a large diversity in which modality and measurement technique to use, a “one size fits all” approach with >12° as an indication for slope-reducing osteotomy in revision ACLR may not be optimal. Therefore, establishing mean PTS values on plain radiographs for the different measurement techniques would lead to a better understanding of the regional knee anatomy and improve clinical evaluation and surgical decision-making.

The purpose of this study was to define the mean PTS value in this Emirati population with knee complaints, measured on long lateral radiographs and defined by the anatomic and mechanical tibial axis for the medial and lateral tibial plateaus.

## Material and methods

This was a retrospective study of 213 consecutive Emirati patients visiting a single center (*International Knee and Joint Centre, Abu Dhabi, United Arab Emirates*) between June and September of 2025. Inclusion criteria were for patients >18 years of age with knee complaints visiting the center for the first time with no previous surgery or fracture in the affected knee. Radiographs of the affected knee, main diagnosis, age, gender, and body mass index (BMI) were recorded. Informed consent was given by all patients, and the study was approved by the institutional review board (*MF3867-2025-2*) and conducted according to the Declaration of Helsinki.

### Radiographic measurements

**Figure 1:**
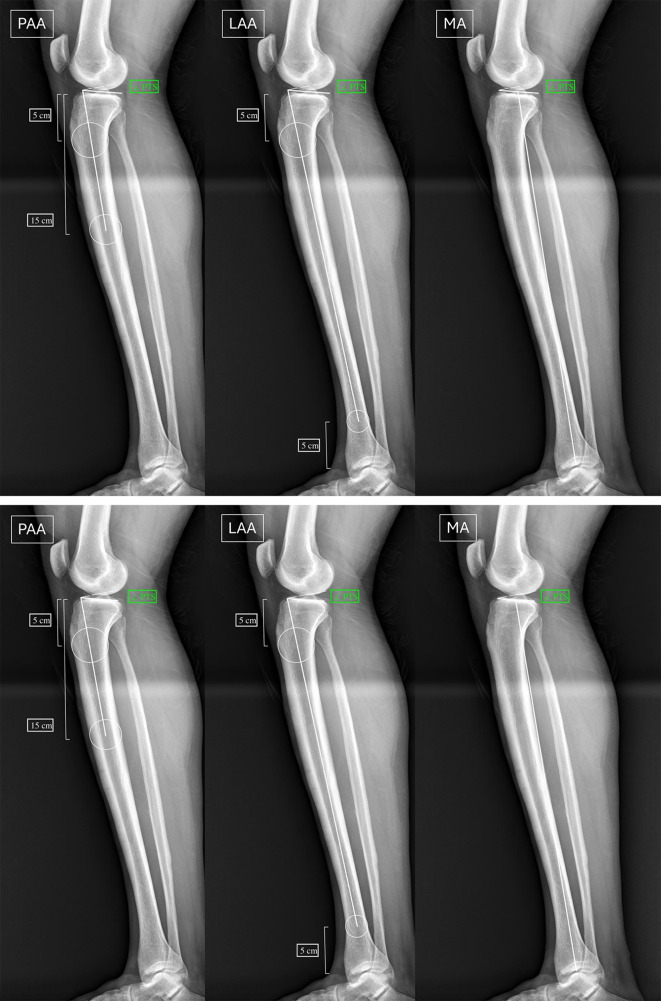
(a) Measurements of medial posterior tibial slope (PTS) using proximal anatomical tibial axis (PAA), long anatomical tibial axis (LAA), and mechanical tibial axis (MA). PTS was measured as the angle between the perpendicular line to the tibial axis and the medial tibial plateau. (b) Measurements of lateral posterior tibial slope (PTS) using proximal anatomical tibial axis (PAA), long anatomical tibial axis (LAA), and mechanical tibial axis (MA). PTS was measured as the angle between the perpendicular line to the tibial axis and the lateral tibial plateau.

Tibial slope was measured on standard long weight-bearing true lateral tibial radiographs with the femoral condyles superimposed at 20 degrees of knee flexion (single leg monopodal stance view). The medial and lateral tibial plateau lines were drawn between the most proximal points of the anterior and posterior tibial plateaus, respectively. The proximal anatomical axis (PAA) line was defined as the line between the anteroposterior tibial midpoint at five and 15 cm distal to the joint line. The long anatomical axis (LAA) line was defined as the line between the anteroposterior tibial midpoint at five cm distal to the proximal joint line and five cm proximal to the distal joint line. The mechanical tibial (MA) axis was defined as the line between the anteroposterior midpoints of both the tibial plateau and the tibial plafond [19]. The PTS angle was the angle between the tibial plateau line and a perpendicular line to the tibial axis (Figures 1a and 1b). To reduce variability in measurements and to mitigate bias, two orthopedic surgeons (*JF and SP*) measured PTS on all radiographs, and the means were used for analysis.

### Statistical analysis

Descriptive statistics were used to evaluate means and standard deviations. For comparisons of PTS values, a paired *t*-test was used, and 95% confidence intervals (95% CI) were reported. Non-paired *t*-tests were used to compare PTS between sexes. The purpose of this study was to establish a reference range for PTS for the Emirati population. To do this, a sample size calculation was performed using the equation *n* = ((Z_α/2_ × SD)/*E*)^2^. Z_α/2_ for a 95% Confidence Interval (CI) = 1.96, Standard Deviation (SD) = 3.5° (common SD in the literature is 3°, we elected to go 0.5° higher), and E (margin of error) was selected to be 0.5°. The sample size calculation required 188 patients. To ensure proper power, >200 patients were set as the sample size. The June to September 2025 timeframe was the first timeframe with >200 patients and was thus selected. Inter-observer reliability was calculated between the two reviewers (*JF* and *SP*) using the Intraclass Correlation Coefficient (ICC); an ICC > 0.90 was considered excellent reliability. Excel (Microsoft) and SPSS v.28.0 (IBM) were used for analysis, and the statistical significance was defined as *p* < 0.05.

## Results

A total of 213 patients, 73 (34%) women and 140 (66%) men, were evaluated with a mean age of 46.0 years (range: 18.8–84.7) and a mean BMI of 30.3 (range: 17.8–47.4). Of the evaluated knees, 98 (46%) were left-sided and 115 (54%) were right-sided, and the main diagnoses are summarized in Table 1.

**Table 1:** Main diagnosis of all 213 patients in the study.

*Diagnosis*	*n*	*%*
Knee joint pain	84	39
ACL injury	16	8
Other ligament injuries	3	1
Meniscus injury	30	14
Muscular injury	8	4
Patellofemoral instability	3	1
Osteoarthritis	69	32

The mean medial PTS was 12.8° ± 2.9° (range: 3.7–19.7°), and the mean lateral PTS was 11.9° ± 3.2° (range: 3.9–20.1°) using the PAA. Using the LAA, the mean medial PTS was 14.7° ± 3.3° (range: 4.8–25.5°), and the mean lateral PTS was 13.8° ± 3.7° (range: 5.1–24.8°). For the MA, the mean medial PTS was 12.7° ± 3.1° (range: 2.7–19.7°) and the mean lateral PTS was 12.2° ± 3.1° (range: 3.8–20.1°) (Table 2). There was excellent inter-observer reliability with all values between 0.944 and 0.958 (Table 2).

**Table 2:** Mean posterior tibial slope (PTS) angle (°) for all 213 patients in the study using both medial and lateral tibial plateau and proximal anatomical axis (PAA), long anatomical axis (LAA), and mechanical axis (MA). Intraclass Correlation Coefficient (ICC) was calculated between the two reviewers.

Tibial axis	Mean° (SD)	ICC
PAA medial	12.8 ± 2.9°	0.944
LAA medial	14.7 ± 3.3°	0.952
MA medial	12.7 ± 3.1°	0.950
PAA lateral	11.9 ± 3.2°	0.953
LAA lateral	13.8 ± 3.7°	0.958
MA lateral	12.2 ± 3.1°	0.950

There was a significant difference when comparing the PTS measured using the LAA with the other measurements, both for the medial and lateral tibial plateaus (all *p* < 0.001). However, when comparing the PTS measured using PAA and MA, there was no significant difference for either the medial (*p* > 0.604) or lateral plateau (*p* > 0.146). Furthermore, when comparing the medial PTS with the lateral PTS, there was a significant difference for all three measurement techniques (all *p* < 0.001) (Table 3). Sex did not influence the PTS values (*p* > 0.05).

**Table 3:** Comparison between the different measurement techniques, proximal anatomical tibial axis (PAA), long anatomical tibial axis (LAA), and mechanical tibial axis (MA), for both medial and lateral tibial plateaus, and for each technique comparison between medial and lateral tibial plateaus. Data presented a *p*-value and 95% confidence interval (95% CI).

Tibial axis	*p*-value	95% CI
PAA vs. MA Medial	0.604	[−0.214 – 0.367]
PAA vs. LAA Medial	<0.001	[−2.153 – 1.587]
LAA vs. MA Medial	<0.001	[−2.19 – 1.703]
PAA vs. MA Lateral	0.146	[−0.625 – 0.093]
PAA vs. LAA Lateral	<0.001	[−2.204 – 1.536]
LAA vs. MA Lateral	<0.001	[−1.922 – 1.286]
PAA Medial vs. Lateral	<0.001	[0.539 – 1.222]
LAA Medial vs. Lateral	<0.001	[0.522 – 1.240]
MA Medial vs. Lateral	<0.001	[0.290 – 0.787]

A subgroup analysis, with patients diagnosed with ACL tear or high-grade osteoarthritis (Kellgren-Lawrence grade 3–4) compared to the rest of the study population, revealed no significant differences in all measurements of slope (*p*-values between 0.213 and 0.794).

## Discussion

The main finding of this study is the establishment of the mean PTS value, 12.7–12.8° for medial and 11.9–12.2° for lateral, in this Emirati population using the most accurate and common ways of measuring PTS [19, 20]. There have been reports that PTS could be higher in some populations, for example, in Saudi Arabia and Nigeria [8, 11]; however, in those studies, the anterior tibial cortex was used as the tibial axis when measuring PTS, which has been shown to result in higher values of around three degrees [21]. A systematic review by Mandalia [20] reported that, using the same measurement technique as we have in this study, the normal PTS value is between six and 12°. Our results demonstrate that the mean Emirati PTS values are higher than those of others and should be considered when using data from other populations for clinical practice.

As a second finding, we show that the MA measurement of PTS is closely related to the PAA measurement, but using the LAA to measure PTS results in significantly higher values and therefore, even though we agree with a previous study showing the MA to be the most accurate measurement of PTS [19], both MA and PAA could be used in a clinical setting to measure the PTS. On the other hand, the LAA should be used with caution for clinical application until further research clarifies cut-off values for this measurement technique [21].

In our study, there was a significant difference between the medial and lateral tibial plateau measurements, consistent with previous findings [22]. Since both an increased medial PTS and an increased lateral PTS are considered risk factors for ACL graft failures, evaluation of a patient with a failed ACL reconstruction should include long tibia radiographs and PTS measurements [5, 23], but whether the medial or lateral PTS is more accurate or better predict ACLR failure is unknown. However, it has been reported that the lateral PTS measured on MRI is the most consistent risk factor among patients with ACL tears [24], but since radiographs are less expensive and more available, and since most studies showing failure rates and long-term outcomes have used the medial PTS, both measurements can be used [5, 20].

Another interesting finding is that 61.5 % of our study population had a PTS > 12° measured with the medial PAA, and since 12° is considered a cut-off value for “high PTS”, this reflects the anatomy and the prevalence of a key risk factor for ACL tear and ACLR failure. The most common indication for a slope-reducing tibial osteotomy is a failed ACL reconstruction with medial PTS >12° measured with PAA.[14] When considering that the mean PTS in the Emirati population with knee complaints is >12°, and possibly even higher in patients with ACL tears, a slope-reducing osteotomy could become a common procedure among revision ACL reconstructions in this region. It is important to note that the indication for slope-reducing osteotomy is multi-factorial, with objective and subjective stability, meniscus status, and reason for previous ACLR failure all playing important roles in the decision-making, not only the PTS value. However, as biomechanical studies have shown, the benefits of reducing the PTS, and thereby the loading forces on the ACL graft [2], and clinical research has shown these procedures to be safe and produce good clinical outcomes [25], no change in surgical threshold is indicated.

The authors recognize some limitations of the study, most importantly that all patients had knee problems and were visiting a knee center for evaluation, and could potentially not represent knees without any abnormality/diagnosis. However, as there were vast differences in the diagnoses, we believe this to be a representative sample. Some strengths of the study are the large sample size, the multiple measurement techniques, and the fact that the patients were collected consecutively.

## Conclusion

In this Emirati population, the mean medial PTS was 12.7–14.7° and the mean lateral PTS was 11.9–13.8° for the differing measurement techniques. These values are higher than other reported populations and can be used as a reference when evaluating knee injuries and contribute to surgical decision-making in ACL-deficient knees.

## Data Availability

The datasets used and analyzed during the current study are available from the corresponding author on reasonable request.
